# Supplementary material for: Deletion of wfs1 Impairs Oligodendrocyte Precursor Cells Dorsal Distribution and Myelination Through the wfs1-hmgcs1 Axis in Zebrafish
Source: Biology (Basel). 2026 Aug 16;15(16):1402. doi: 10.3390/biology15161402 (PMC13510151; doi:10.3390/biology15161402)
Supplement: Supplementary file 1 [file biology-15-01402-s001.zip › File S1-Transcriptome sequencing data and differentially expressed genes (DEGs) in wfs1-- zebrafish at 5 dpf/1.GO/wfs1KOvsWT/DOWN/wfs1KOvsWT.down_GOdot.pdf]

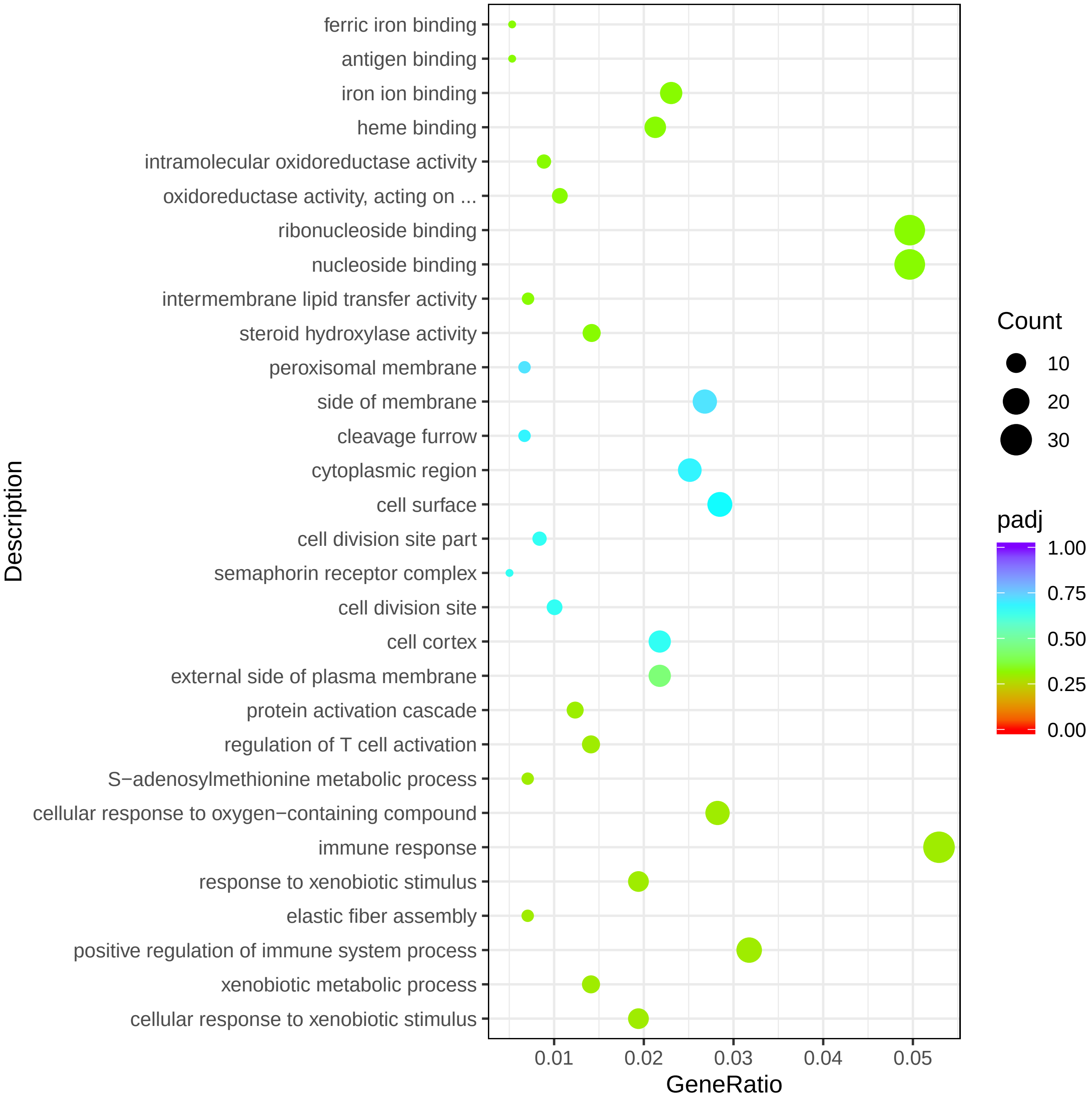

ferric iron binding
antigen binding
iron ion binding
heme binding
intramolecular oxidoreductase activity
oxidoreductase activity, acting on ...
ribonucleoside binding
nucleoside binding
intermembrane lipid transfer activity
steroid hydroxylase activity
peroxisomal membrane
side of membrane
cleavage furrow
cytoplasmic region
cell surface
cell division site part
semaphorin receptor complex
cell division site
cell cortex
external side of plasma membrane
protein activation cascade
regulation of T cell activation
S−adenosylmethionine metabolic process
cellular response to oxygen−containing compound
immune response
response to xenobiotic stimulus
elastic fiber assembly
positive regulation of immune system process
xenobiotic metabolic process
cellular response to xenobiotic stimulus
Description
0.01
0.02
0.03
0.04
0.05
GeneRatio
Count
10
20
30
padj
1.00
0.75
0.50
0.25
0.00
